# A School-Based Five-Month Gardening Intervention Improves Vegetable Intake, BMI, and Nutrition Knowledge in Primary School Children: A Controlled Quasi-Experimental Trial

**DOI:** 10.3390/nu17193133

**Published:** 2025-09-30

**Authors:** Nour Amin Elsahoryi, Omar A. Alhaj, Ruba Musharbash, Fadia Milhem, Tareq Al-Farah, Ayoub Al Jawaldeh

**Affiliations:** 1 Department of Nutrition, Faculty of Pharmacy and Medical Sciences, University of Petra, Airport Road, Amman 11196, Jordan; omar.alhaj@uop.edu.jo (O.A.A.); fadia.milhem@uop.edu.jo (F.M.); 2 Ruba Musharbash Center for Nutrition Counseling, Amman 910771, Jordan; rubaresearch20@gmail.com; 3 Tareq Al-Farah Center for Nutrition Counselling, Al-Fuhies 19152, Jordan; tarekalfarah63@hotmail.com; 4 Regional Office for the Eastern Mediterranean (EMRO), World Health Organization (WHO), Cairo 11371, Egypt

**Keywords:** school gardening intervention, nutrition education, vegetable intake, body mass index (BMI), childhood obesity, knowledge attitudes practices, Jordan, Middle East, primary school children

## Abstract

**Background/Objectives**: Childhood obesity rates in Jordan have reached alarming levels, with 28% of school-age children classified as overweight or obese. School-based gardening interventions show promise for promoting healthy eating behaviors, yet limited research exists in Middle Eastern contexts. This study evaluated the effectiveness of a five-month school-based vegetable gardening and nutrition education intervention on anthropometric measures, dietary intake, and knowledge, attitudes, and practices (KAP) regarding vegetable consumption among Jordanian primary school children. **Methods**: A quasi-experimental controlled trial was conducted with 216 students (ages 10–12 years) from two demographically matched schools in Amman, Jordan. The intervention group (*n* = 121) participated in weekly one-hour gardening sessions combined with nutrition education and vegetable tasting activities over five months, while the control group (*n* = 95) continued the standard curriculum. Outcomes measured at baseline and post-intervention included anthropometric assessments, dietary intake via 24 h recalls, and vegetable-related KAP using a validated questionnaire. Data were analyzed using paired *t*-tests and repeated measures ANCOVA. **Results**: The intervention group demonstrated significant improvements in body composition, including reductions in BMI (−1.57 kg/m^2^), weight (−1.88 kg), and BMI z-score (−0.37), while controls showed minimal increases. Vegetable intake showed significant time × group interaction (*p*-value = 0.003), with a non-significant increase in the intervention group (2.7 to 2.9 times/day) and a non-significant decrease in the controls (2.5 to 2.4 times/day). Dietary quality improved, including increased fiber intake (+2.36 g/day) and reduced saturated fat consumption (−9.24 g/day). Nutrition knowledge scores increased substantially in the intervention group (+22.31 points) compared to controls (+1.75 points; *p*-value ≤ 0.001). However, attitudes and practices toward vegetable consumption showed no significant changes. **Conclusions**: This intervention effectively improved body composition, dietary quality, and nutrition knowledge among Jordanian primary school children. These findings provide evidence for implementing culturally adapted school gardening programs as childhood obesity prevention interventions in Middle Eastern settings, though future programs should incorporate family engagement strategies to enhance behavioral sustainability.

## 1. Introduction

Childhood obesity has been termed as one of the major public health concerns of the twenty-first century [1]. As of 2022, over 390 million children and adolescents aged 5 to 19 years old were found to be overweight, with a subset of 160 million found to be obese, about 20 percent of the total number of children and adolescents in this age group worldwide (compared to 8 percent in 1990). Health outcomes in both the short and long terms are profoundly impacted by this prevalence [2]. The Jordan National Micronutrient and Nutritional Survey (2019) offers nationally representative estimates that show that among children of school age (6–12 years old) in Jordan, 16% of the population is overweight and 12% is obese, giving an overall prevalence of 28% using WHO 2007 BMI-for-age cutoffs [3]. This trend has been most prominent in the developing world, where children are exposed to high-fat, high-sugar, high-salt, energy-rich, and micronutrient-poor foods at the same time as experiencing nutritional inadequacies during important periods of development [2]. Childhood obesity has far-reaching implications that go beyond body health to include increased susceptibility to non-communicable diseases [4], poor psychosocial outcomes, and adoption of unhealthy eating habits that continue to affect the individual later in life [5,6]. Prevention is therefore important; early intervention can prevent long-term health problems that may arise later in life [6,7,8,9].

Eating behaviors in childhood and adolescence, including low fruit and vegetable consumption and high consumption of processed foods and sugar-sweetened beverages [10,11], are both a foundation of the pathophysiology of obesity and its persistence [1,12]. Even though the literature has set a clear guideline on the number of fruits and vegetables to be consumed daily, most children do not reach this requirement, and only about 20% of children consume the recommended number of fruits and vegetables [12]. The gap is more significant among minority groups and low-income groups [13,14].

According to recent regional surveys, children and adolescents in the Middle East eat much less fruit and vegetables than the amounts recommended in global guidelines and are notably dependent on high-energy foods with low nutrient content [15,16]. Such a nutritional gap highlights the need to introduce culturally modified interventions. It has been empirically revealed that low consumption of vegetables is associated with higher likelihood of obesity, metabolic disorder, and poor overall diet quality, and consuming more sugar-sweetened drinks and fast food significantly contributes to the likelihood of being overweight and obese (by 1.20 and 1.17, respectively) among children and adolescents [17,18]. The acquisition of dietary preferences and eating habits is an important aspect in child development because the established patterns typically continue into adulthood [19]. Therefore, the elementary school age is a critical period to implement effective interventions to promote healthy eating habits and interfere with the process of developing obesity and chronic diseases [20,21].

Schools offer the best setting to deliver comprehensive nutrition programs as they have a large population of children with diverse socioeconomic statuses, can ensure the repetition of exposure to healthy foods, and offer structured settings that allow the systematic introduction of academic and behavioral practices [22,23]. Such interventions in schools, as supported by systematic reviews, are among the most effective measures in promoting dietary behaviors and preventing obesity, especially when combined with education and implementing environmental changes [1,24,25,26]. The empirical research shows that multi-component programs that combine educational, environmental, and behavioral components lead to significant changes in the dietary habits of children [22,27,28] and can thus target obesity-related outcomes more effectively than single- or dual-plane educational or environmental programs [29]. The results indicate that school-based nutrition programs increase knowledge regarding healthy eating, positively change attitude to fruits and vegetables [30,31], influence dietary quality by increasing the score on scales like the Healthy Eating Index, and, on some occasions, produce desirable changes in anthropometric measures, including body mass index (BMI) and waist circumference [32,33]. Additionally, schools are natural laboratories where scaling and sustaining evidence-based interventions [34,35] are possible, so they are essential venues in which to deal with the multifactorial etiology of childhood obesity, as well as promote academic and overall child development [23,36].

Gardening programs have been considered one of the most promising school-related interventions in terms of improving eating habits among children as well their relationship with food, especially regarding vegetables. School-wide gardening projects of this kind, such as the TX Sprouts program in the United States, have shown statistically significant improvements in vegetable intake and dietary fiber intake, with no associated reduction in the BMI, among elementary school students [1]. However, the effectiveness of similar culturally specific interventions in the Middle Eastern setting is under researched [1,7,25,37]. The premise of garden-based interventions is that firsthand, hands-on experience with the growing, harvesting, and preparing of vegetables [38] increases familiarity, desire to eat [39,40], and subsequent liking of these foods—mechanisms that directly overcome the obstacles of food neophobia and lack of exposure to vegetables known to underlie poor vegetable consumption patterns among children [40,41,42]. Systematic reviews and meta-analyses also point to the fact that gardening interventions have the potential to increase the levels of fruit and vegetable consumption, nutrition knowledge, and positive attitude to healthy foods, and some studies report positive changes in body composition indicators (BMI and waist circumference) [7,43]. The success of these programs is enhanced when nutrition education and cooking elements are combined [1,38] to bring about a complete practice that involves several determinants of dietary behavior, such as knowledge, attitudes, competencies, and direct sensory exposure to nutritious foods [40,44,45,46]. The currently available studies also show that school garden programs are especially effective among elementary-school-aged children, with significant increases in vegetable preference levels, readiness to taste vegetables [47,48], and objective consumption rates reported as the outcomes. This fact further indicates that elementary school children are an ideal population in terms of school-garden-based nutrition interventions [49,50].

The evidence that school-based garden programs can have beneficial effects on the diets and health outcomes of children is increasing, but a set of methodological shortcomings undermines our ability to draw generalizations about the findings as a whole. The limitations of previous studies often include their short intervention periods, small sample sizes, poor control groups, and their use of self-reported data regarding diet rather than full objective assessments. Moreover, most studies have been performed in higher-income, mainly white populations, which limits the extrapolation of the results to other student groups, such as those with low socioeconomic status, who are at higher risk of obesity and poor diet quality. The current literature also does not provide much information regarding the optimal length and intensity to induce meaningful and lasting dietary and health outcomes, with most studies evaluating relatively short-term interventions that may not provide a long enough exposure to induce a lasting behavioral change [40]. Although the positive effects on knowledge and attitudes are prevalent [31,40], the effects on dietary intake and physiological measures have been less documented, and more effective evaluation strategies are required to measure the entire scope of the intervention effects and clarify the mechanisms through which the intervention may have health and developmental outcomes in children [51]. There are research gaps in the study of body composition measures, dietary intake, and knowledge, attitudes, and practice measures of vegetable consumption in students between the fourth and sixth grades. To date, Jordan has not conducted any controlled trials that fully examine the impact of gardening interventions on anthropometric measurements, dietary intake, and vegetable-related knowledge, attitudes, and practices in children. The concise research gap will provide contextually relevant information for future policy formulation in school health.

Therefore, the present study was designed to evaluate the effectiveness of a five-month school-based vegetable gardening and education intervention on body composition, dietary intake, and knowledge, attitudes, and practices (KAP) regarding vegetable consumption in 4th–6th-grade students. The study compared these outcomes between the intervention group and a control group, hypothesizing that students participating in this intervention would show improvement in their body composition, dietary intake, and KAP regarding vegetable consumption.

## 2. Materials and Method

### 2.1. Study Design and Participants

This study was a quasi-experimental, controlled trial undertaken on two demographically matched primary schools in Amman, Jordan, in order to consider the efficacies of a school-based vegetable gardening program and a five-month supportive educational program on children’s anthropometric measurements, their dietary intake, and KAP regarding vegetable consumption. Schools were selected for this research based on the uniformity of their geographic area and similarity of features including demographics, socioeconomic status, and size; this was done to reduce confounding variance in the study. The intervention was performed during a complete school term (not including public holidays), and the same pre- and post-intervention tests were conducted in both schools. Considering that one of the institutional sites was exposed to the intervention and the other acted as control, the site variable confounded the assignment of participants. Therefore, the findings represent the combined effect of site and intervention; any inferences between groups are limited by institutional differences that were not offset by matching.

Data collection occurred at two time points: baseline (August 2024) and follow-up (January 2025, immediately after the 5-month intervention). Both intervention and control groups were assessed at these same time points to allow for valid pre–post comparisons.

Students in grades 4–6 (aged 10–12 years) were eligible for participation in this study. Complete study information was formally distributed to all parents through school administration prior to consent collection, and written, signed informed consent was obtained from parents, along with written or verbal assent from participating students before study initiation. The inclusion criteria required written parental consent, child assent, and minimum 80% school attendance during the study period.

Children were excluded from participation if they presented with high-level food allergies or restrictive diets that could interfere with vegetable tasting activities, diagnosed eating disorders, medical conditions affecting normal growth patterns, or current enrollment in other nutrition intervention programs. Additionally, students unable to participate in outdoor gardening activities or additional school-based activities beyond the routine curriculum were excluded, as were those with environmental allergies including pollen allergies, dust allergies, or respiratory disorders that would prevent safe participation in gardening activities.

One school was assigned to the intervention group and received structured weekly sessions combining practical gardening activities, nutrition education, and vegetable tasting over the five-month intervention period. The control school served as a natural control comparison by continuing with their standard curriculum without exposure to gardening activities, nutrition education sessions, or vegetable-tasting instruction, ensuring that any observed differences could be attributed to the intervention rather than external factors.

The three main outcomes were collected at baseline and post-intervention, including anthropometric information (height and weight); dietary intake, identified by means of a 24 h dietary recall; and vegetable-related KAP, estimated based on an 18-item questionnaire adapted from the existing literature [52,53].

### 2.2. Ethics Consideration

The Institutional Review Board at the University of Petra provided ethical approval (Approval Number: E/H/3/5/2024, decision date: 30 May 2024), and the research departments of the involved school districts also provided ethical approval. Each parent or guardian provided a written informed consent form, and all the participating students provided verbal or written assent prior to the data collection process. The reporting of the results of this quasi-experimental study follows the TREND (Transparent Reporting of Evaluations with Nonrandomized Designs) Statement [54] in order to follow the scientific transparency and rigor of methods for research. This guideline is specific to nonrandomized interventions and allows sufficient reporting about the context and technical content of the intervention and its implementation and results.

### 2.3. Sample Size Calculation

The present quasi-experimental school-based intervention was a priori designed to consider sample-size calculation with G*Power version 3.1.9.7. The repeated measures ANCOVA (within–between interaction) was been chosen as the correct statistical test to evaluate the effects of time × group that will be measured at two points (pre-intervention and post-intervention) in two independent groups (intervention and control). The computation used a moderate effect size (f = 0.25), a Type I error probability (α) of 0.05, a desired statistical power (1 − β) of 0.95, and an expected correlation between repeated measures of 0.5. The estimated minimum required total sample size was 54 participants (27 in each group). On the one hand, a significantly higher sample size was targeted to strengthen the statistical robustness and account for possible dropouts, as well as to gain higher external validity. In such a way, 300 students (150 students at each school) were sampled, and all of them were enrolled for the baseline assessments. This sample size exceeds the minimum sample size requirement and is similar to or higher than those used in similar school-based diet and gardening interventions; thus, this sample size gives the study sufficient power to detect effects of interaction between the groups and time on behavioral and anthropometric measures.

### 2.4. Description of the Gardening Intervention

The intervention was based on the social–ecological model that envisions a child as an inhabitant of the non-autonomous systems that are interdependently related to one another and which, over time, shape the health-related behavior of the child [55]. The gardening component became a primary stage to transform this theoretical framework into practice, with the focus on experiential learning, interaction with the environment, and skill development in the school setting. To conduct the intervention, an outdoor teaching garden that was 1000 square meters in size was built on land owned by the intervention school in Amman, Jordan, at the start of the spring semester before the initial period of data collection. The garden infrastructure was based on the TX Sprouts model [6], with self-irrigating raised beds that contained indigenous herbs and vegetables and a separate storage shed to store tools and teaching materials. To facilitate the work, the school received the necessary gardening equipment, such as rakes, watering hoses, benches, gardening gloves, and composting bins, as well as educational material, tables, whiteboards, portable handwashing stations, and basic cooking instruments. Crops were selected according to seasonal suitability, soil suitability, and cultural suitability, which means that no unusual or bizarre crop was included in the garden and that the garden contained mostly vegetables commonly eaten in the local Jordanian diet [15,56]. The crops were lettuce, bell pepper, tomato, cucumber, and native herbs. During the intervention (five months), the students received a one-hour gardening lesson every week from trained facilitators. The classes had a follow-up curriculum that involved simple aspects regarding horticulture, including soil preparation, planting, weeding, pest control, and harvesting. The planting and growing involved direct interaction with each child, who gained first-hand experience of how the food systems worked and seasonal changes as the crops grew. The gardening intervention focused on experiential learning and skill development through direct interaction with food production processes, which was designed to reduce food neophobia and increase vegetable acceptance through repeated exposure. The gardening aspect was, therefore, not only an agricultural factor but also a pedagogical one, and helped build life skills, health literacy, and behavior change as part and parcel of a child-focused, culture-appropriate mode.

### 2.5. Description of the Education Intervention

One-hour culturally adapted nutrition education sessions were provided to Arabic-speaking students on a weekly basis over the course of 20 weeks (five months), immediately after the gardening sessions. This adaptation included translation to Arabic, culturally appropriate crop selection (lettuce, bell peppers, tomatoes, cucumber, native herbs), and educational materials designed for the Jordanian context with locally relevant examples and imagery. The sessions were conducted by professionals trained in child-oriented nutrition education and behavioral modification. The curriculum adhered to KAP frameworks in that it was based on behavior change theories, such as social cognitive theory and experiential learning models, which have been confirmed in previous school-based interventions [26,49,52]. Each session was based on key nutrition themes, such as the benefits of vegetables in promoting good health, the need for dietary diversity, the reciprocation of meals, and the need to make healthy lunch boxes. The most important trait of the educational approach was the interaction mode of delivering the content, which encompassed the combination of the narrative, learning games, group discussions, and live demonstrations that have been proven in previous studies to enhance the levels of engagement and retention in school-age children [57,58].

In order to ensure the reinforcement of the messages, both in terms of a visual and emotional form, each session included the use of colorful, cartoon-like posters that were uniquely illustrated by the research team (in cooperation with a senior educational social specialist to ensure age/gender appropriate pedagogy) to ensure nutritional information accuracy. These posters presented one new vegetable a week and depicted a picture of its nutritional worth in the form of playful, age-appropriate characteristics. An illustrative example is provided in the Supplementary Materials (Supplementary Files S1, as well as a set of texts sent to the children during the study. Notably, a guided tasting process was also conducted in each session to ensure that students had a taste of some uncooked or little-cooked vegetables covered in the lesson. Through guided tasting, the repeated exposure to sensations was implicitly performed as an intervention strategy with clear evidence in the field of behavioral nutrition regarding the reduction of food neophobia and the increase in vegetable acceptance [1,59]. The vegetables used in tasting were seasonally appropriate and consistent with the output of the corresponding gardening component, which itself promoted correlation between the theoretical and practical activities. The tasting component was not only experiential but also behavioral; the message being that the vegetables were not only good but also good to eat. The nutrition classes took place in classrooms next to the school garden and were highly interwoven with the gardening plan, so the children could relate what they had just planted and grown with what they studied during the nutrition classes. The two focal points of knowledge transfer and positive food impression were meant to develop cognitive comprehension as well as emotional consistency related to healthy eating. All the materials, posters, tasting procedures, and scripts of the activities were culturally adjusted to the Jordanian setting and piloted by educational professionals with the goals of determining their appropriateness to the age group and pedagogical efficiency.

### 2.6. Outcome Measurements

To evaluate the effectiveness of the school gardening intervention for the age groups proposed in the study, multiple outcomes were measured at two time points: intervention initiation and post-intervention. The outcomes were measured before and after the intervention and included (1) anthropometric measurements—height, weight, derived BMI, BMI-for-age z-score, and percentile; (2) dietary intake through repeated 24 h recalls; and (3) vegetable-intake-related knowledge, attitudes and practices (KAP), measured using a validated and structured questionnaire consisting of 18 items based on previous interventions conducted with schoolchildren [52,53].

### 2.7. Anthropometric Measurements Assessment

Height, weight, and BMI, as the anthropometric measures, were taken at the baseline and post-intervention stages with standardized procedures, as defined by World Health Organization (WHO) [60]. A portable stadiometer Seca 213 (Seca 213, seca GmbH & Co. KG, Hamburg, Germany) was used to measure height to the nearest 0.1 cm; weight was measured to the nearest 0.1 kg using a calibrated digital scale (Tanita HD-351, Tanita Corp., Tokyo, Japan). Participants were required to wear light clothes and no shoes. Every measurement was repeated twice and averaged out to be accurate; when differences in the measurements of height and weight were noticed to be more than 0.5 cm or 0.2 kg, respectively, a third measurement was taken. BMI was estimated by dividing weight in kilograms by the height in meters squared (kg/m^2^). BMI-for-age z-scores and percentiles were computed to take into consideration age-specific and gender-specific variation in growth using the WHO 2025 growth standard [60] for children aged between 5 and 19 years through WHO AnothroPlus software (version 1.0.4). Using this allows classifying nutritional status using standardized cutoffs, namely underweight (z < −2 SD), normal weight (−2 SD ≤ z ≤ +1 SD), overweight (z = +1 SD < z = +2 SD), and obese (z = +2 SD). The field researchers performing all measurements were trained, and a reliability test was performed on them before data were collected. The measurement facilities were established in the private classrooms of the school, so that confidentiality was guaranteed and anxiety among the schoolchildren was minimal. The equipment was calibrated on a weekly basis and every piece of data was entered as soon as possible in locked electronic records to reduce transcription mistakes. The anthropometric assessment protocol is similar to that used in previous studies investigating other school-based interventions, such as TX Sprouts [1,42], with WHO z-scores and percentiles being utilized as standardized measures of growth and intervention effects using primary school children.

### 2.8. Collection of Dietary Data via 24 h Dietary Recalls

In order to investigate the dietary intake of the participants, 24 h dietary recalls were conducted at baseline and after the intervention. These recalls were unannounced and administered within a one-week window. One weekday and one weekend were assessed in the recalls to account for intra-individual variability in eating patterns. Follow-up was conducted to minimize missing data and response bias in cases of student unavailability or refusal cooperate. Dietary intake was measured on two independent 24 h dietary recalls, one of them on a weekday and one on a weekend, implemented without prior notice and with a time period of one week during both the post-intervention and baseline phases. To improve the accuracy and completeness, the dietary recalls were conducted based on the USDA Automated Multiple-Pass Method (AMPM), a validated five-step, multi-pass interview procedure for pediatric populations [61]. The recall sessions took 20–30 min each. In an attempt to facilitate the estimation of portions sizes, the participants received a food portion booklet before the data were collected. This tool was used and exercised on the education intervention sessions and applied by the participants in the recall process. Other strategies that were introduced sought to enhance the accuracy of intake reporting. The parents were urged to peruse their child’s food recall entries, especially regarding the kind of food and its estimated portion size. Food Processor nutrition analysis software (ESHA, version 11.15), purchased in February 2025 was used to assess the 24 h recalls, as it has a large food database and improved nutrient calculation algorithms. Standardized recipes comprised local composites. Where Jordanian foods were not available in the ESHA database (v11.15), nutrient profiles were built with the label data or nearest equivalents.

Collection of the data in the current study was performed under the supervision of trained nutritionists who held either a clinical or a bachelor’s degree in nutrition and had a minimum of six months experience in dietary assessment. All of the investigators were structurally trained on data collection before the real-time data collection team collected the data. Inter-rater reliability procedures were used to ensure uniformity with the different interviewers. Dietary components to be analyzed were selected by following the public health relevance and evidence-based nutritional priorities determined by the Dietary Guidelines Advisory Committee (DGAC) [62]. To determine the overall dietary balance, macronutrients such as the total energy intake and the proportion of energy, in terms of the carbohydrates, fats (including saturated fats), and proteins, were considered. Micronutrients were also classified as shortfall nutrients (e.g., vitamins A, E, C; magnesium, iron), nutrients of public health concern (e.g., dietary fiber, calcium, vitamin D, and potassium), or nutrients to be reduced (e.g., saturated fat, sugars, and sodium). Micronutrients of public health concern were defined, according to the Dietary Guidelines for Americans, as nutrients that are under-consumed relative to recommendations and are associated with health concerns when intake is inadequate, specifically including dietary fiber, calcium, vitamin D, and potassium in children. These groups exhibit an inadequacy that is highly related to public health concerns involving children. These particular nutrients were selected in accordance with the objectives of the intervention, which were to encourage more healthy eating habits based on experience and repetitive exposure to vegetables. The current piece of work allows assessing changes in terms of nutrient intake through comparisons between pre- and post-intervention measures, which elucidates the nutritional outcomes of the school-based gardening and education program.

### 2.9. Nutritional Knowledge, Attitudes, and Practices (KAP) Towards Vegetable Intake

A semi-structured (KAP) questionnaire was applied to study the cognitive and behavioral aspects concerning the consumption of vegetables by students. In the current investigation, questionnaires were created by modifying the existing, validated questionnaires used in previous nutrition interventions in schools [52,53]. Validation of the instrument was performed based on the Jordanian schooling context, simplifying language use, making it culturally relevant, and making sure that the types of vegetables included in the instrument are available both in the school and the local market. The 18-item final version of the KAP tool was designed to be dichotomous (Yes = 1, No = 0), with equal distribution among three domains—knowledge, attitudes, and practices (6 items each). The knowledge section included basic nutritional information, as well as commonly held beliefs; the attitudes section revealed students’ opinions on the use of vegetables in terms of taste, health value, and preference; and the practices section recorded self-reported daily vegetable intake in both main meals and snacks. Language and conceptual equivalence were subsequently tested by using a forward–back translation process, as proposed by Khalaila (2013) [63], after a process of initial translating the version into Arabic. Validity of content was established by examining the item with six nutrition education and school health experts, and it achieved validity index of content level (CVI) of 1.00 and validity index of scale level (S-CVI/Ave) of 0.99. The mean score of semantic equivalence was 3.56 out of 4, which means a high level of conceptual consistency. Before data collection, the clarity of instructions and face validity were tested in a pilot study undertaken with five students. Internal consistency (Cronbach’s α) was measured on baseline data (*n* = 40) and test–retest reliability in a subsample (*n* = 40, 10-day interval). Construct validity was assessed with principal component analysis (PCA) (varimax rotation) after confirming sampling adequacy with the Kaiser–Meyer–Olkin measure and Bartlett’s test of sphericity. The internal reliability was acceptable (Cronbach alpha = 0.728); the test–retest test demonstrated high temporal reliability (r = 0.78, *p*-value ≤ 0.001). PCA provided construct validity and supported that a three-domain structure (in line with previous theoretical models) is appropriate [64]. To sum up, it has been identified that the adapted KAP tool was psychometrically sound and appropriate to assess cognitive and behavioral measures of vegetable consumption amounts among Arab-speaking primary school students that took part in garden-based nutrition programs.

### 2.10. Statistical Analysis

IBM SPSS statistics version 28 was utilized to perform the statistical analyses. To summarize the baseline characteristics, descriptive statistics (means, standard deviations, and percentages) were computed. Independent sample *t*-tests were utilized to assess the differences in the baseline continuous variables between the intervention and control groups, whereas the chi-square test was utilized to evaluate the differences in the baseline categorical variables (*p*-value < 0.05, two-tailed). Per-protocol analysis, including the subjects who attended the baseline and post-intervention measurements (*n* = 121 in the intervention school; *n* = 95 in the control school), was performed. Listwise deletion was used as a method of handling missing data. Before analysis, the normality assumption was verified through Shapiro–Wilk tests and histogram measures. Since the outcome variable was not normally distributed, a log-transformation was performed before modeling; further analysis of the model residues was performed to verify the assumptions. The original scale is used to report the results so that they can be easily interpreted. In order to determine the changes within each group over time, paired-sample *t*-tests were performed within groups for pre–post changes. Analysis of covariance (ANCOVA) was used to compare between-group post-intervention outcomes, where associated baseline values were adjusted. The models included covariates of age (in months), gender, and grade (4–6). The core outcomes included the anthropometric factors (e.g., BMI-for-age z-score), the dietary intake variables (e.g., total energy intake, macronutrient distribution, particular micronutrient intake), and knowledge, attitudes, and practices regarding the consumption of vegetables. In the case of BMI-for-age Z-score and percentile, only the baseline value and grade were adjusted, as the WHO z-scores are already age- and gender-adjusted. A two-tail test was applied, and statistical significance was set as *p*-value < 0.05. De-identified data contained unique study IDs, and the data were only stored on secure and encrypted servers with limited access. To address baseline imbalances in gender distribution and height between groups, all primary analyses were conducted using repeated measures ANCOVA with appropriate covariate adjustments, including age, gender, and grade. For anthropometric outcomes (BMI z-score and percentile), analyses were adjusted for baseline values and grade only, as WHO z-scores are inherently age- and gender-standardized.

## 3. Results

### 3.1. Baseline Characteristics of the Study Participants

The baseline characteristics of participants in the intervention (*n* = 121) and control (*n* = 95) groups were compared to assess initial equivalence, as shown in Table 1. The total sample of 216 children had a mean age of 10.18 ± 1.22 years, with no statistically significant difference between groups (control: 10.35 ± 1.17 vs. intervention: 10.05 ± 1.24; *p*-value = 0.073). Gender distribution differed significantly between groups (*p*-value ≤ 0.001), with males representing 25.0% (*n* = 22) of the control group compared to 75.0% (*n* = 66) of the intervention group, while females showed the opposite pattern (57.0%, *n* = 73 control vs. 43.0%, *n* = 55 intervention). Weight measurements showed no significant group difference (total: 42.17 ± 2.62 kg; control: 41.80 ± 3.12 kg vs. intervention: 42.45 ± 2.11 kg; *p*-value = 0.072). However, height differed significantly between groups (*p*-value ≤ 0.001), with control participants being taller (133.64 ± 5.07 cm) than intervention participants (130.32 ± 2.94 cm). These findings indicate some baseline imbalances between groups, particularly in gender distribution and height, which should be considered in subsequent analyses of intervention effects.

### 3.2. Anthropometric Outcomes

Table 2 shows that the gardening intervention led to significant differences in several anthropometric outcomes between the control and intervention groups. For height, both groups experienced statistically significant increases in the mean post-intervention. The control group increased from 133.64 ± 5.07 cm to 134.92 ± 4.93 cm (*p*-value ≤ 0.001; Δ = +1.28 cm), and the intervention group increased from 130.32 ± 2.94 cm to 131.61 ± 3.16 cm (*p*-value < 0.001; Δ = +1.29 cm). However, there was no significant difference between groups (*p*-value = 0.941), although a significant group × time interaction was observed (*p*-value < 0.001).

**Figure 1:**
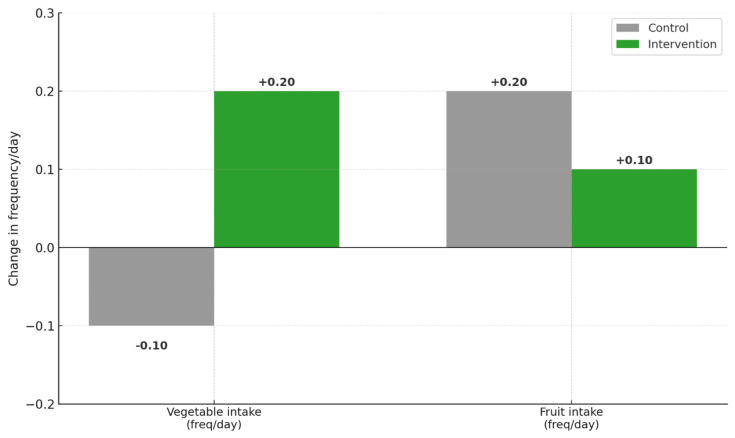
Change in vegetable and fruit intake frequency (servings/day) after the gardening intervention among primary school children in the control and the intervention groups.

For weight, there was a significant time × group interaction. The control group’s weight increased from 41.81 ± 3.12 kg to 42.67 ± 3.06 kg (*p*-value ≤ 0.001; Δ = +0.86 kg), while the intervention group’s weight decreased from 42.45 ± 2.11 kg to 40.57 ± 2.14 kg (*p*-value ≤ 0.001; Δ = −1.88 kg). In terms of BMI (kg/m^2^), the control group exhibited a slight increase from 23.47 ± 2.07 to 23.50 ± 1.99 (*p*-value ≤ 0.001; Δ = +0.03), whereas the intervention group had a meaningful reduction from 25.02 ± 1.46 to 23.45 ± 1.45 (*p*-value ≤ 0.001; Δ = −1.57). The group effect was statistically significant (*p*-value = 0.001). The BMI z-score followed a similar trend. The control group increased from −0.45 ± 1.08 to 0.02 ± 1.17 (*p*-value = 0.050; Δ = +0.47), while the intervention group decreased from 0.36 ± 0.76 to −0.01 ± 0.85 (*p*-value ≤ 0.001; Δ = −0.37). Finally, for BMI percentile, the control group showed a small and non-significant increase from 96.29 ± 6.16 to 96.51 ± 4.71 (*p*-value = 0.072; Δ = +0.22), while the intervention group showed a significant decrease from 97.89 ± 1.16 to 97.10 ± 2.16 (*p*-value = 0.002; Δ = −0.79). A significant group effect (*p*-value = 0.031) and significant interaction (*p*-value = 0.002) were also observed for BMI percentile. Frequency/day of vegetable and fruit consumption was assessed pre- and post-intervention to indicate the effects of the school gardening program. In the intervention group, there was an remarkable upswing in vegetable consumption (2.7 ± 1.6 to 2.9 ± 1.5) to 3.1 1.6 times/day, with an absolute change of +0.2; *p*-value = 0.49, whereas in the control group, there was a considerable decline in vegetable consumption (2.5 ± 1.4 to 2.4 ± 1.5 times/day), with an absolute change of −0.1; *p*-value 0.886. There was a strong interaction in terms of time × group; *p*-value = 0.003. The gardening program, therefore, helped boost the consumption of vegetables, as shown in Table 2. However, fruit intake showed minimal non-significant changes in both groups; hence, fruit consumption was not affected. These findings suggest that the school gardening program was more effective in promoting vegetable consumption than fruit consumption among primary school. Regarding the daily consumed servings of fruit and vegetables after this intervention, Figure 1 illustrates that the intervention group showed a positive change of +0.20 servings/day for vegetable intake, while the control group decreased by −0.10 servings/day. For fruit intake, both groups showed modest positive changes (+0.10 servings/day for the intervention group and +0.20 servings/day for the control group), though these changes were not statistically significant. The contrasting patterns between groups demonstrate the intervention’s specific effectiveness in promoting vegetable consumption among primary school children.

### 3.3. Nutrient Intake Outcomes

Table 3 presents the effects of the gardening intervention on children’s intake of macronutrients, shortfall micronutrients, micronutrients of public health concern, and nutrients to reduce. At baseline, average energy intake was 1519.72 ± 398.35 kcal for the control group and 1622.34 ± 450.71 kcal for the intervention group. Post-intervention, energy intake significantly increased in the control group (mean = 1571.97 ± 488.64 kcal, *p*-value ≤ 0.001) and decreased in the intervention group (mean = 1476.99 ± 421.27 kcal, *p*-value ≤ 0.001), with a statistically significant time × group interaction (*p*-value ≤ 0.001). Protein intake decreased significantly within the control group (*p*-value ≤ 0.001) and showed a non-significant decrease in the intervention group (*p*-value = 0.098). A significant group effect was observed (*p*-value = 0.006), though the time × group interaction was not significant (*p*-value = 0.553). Carbohydrate intake decreased in both groups, but more substantially in the intervention group (−14.89 g/day, *p*-value = 0.015), without a significant time × group interaction. Fat intake increased in the control group and decreased in the intervention group, with a significant time × group interaction (*p*-value = 0.025).

Regarding shortfall micronutrients, vitamin A intake increased in both groups without reaching significance. Vitamin E intake significantly increased in the intervention group (*p*-value ≤ 0.001), while it declined in the control group. Magnesium intake significantly improved in the intervention group (*p*-value ≤ 0.001) but declined slightly in the control group. No significant differences were observed in iron or vitamin C intake. For micronutrients of public health concern, fiber intake decreased in the control group (−1.24 g/day) but increased (+2.36 g/day) in the intervention group, with a significant time × group interaction (*p*-value = 0.017). Calcium and vitamin D intake, although not reaching significance, increased post-intervention in the intervention group only. Potassium intake declined in the control group and increased in the intervention group, with a significant time × group interaction (*p*-value = 0.023). In terms of nutrients to reduce, sugar intake decreased in both groups, with no significant time × group interaction. Sodium intake increased in both groups, more so in the control group (+461.93 mg/day), with a significant group effect (*p*-value = 0.010) but no significant time × group interaction. Saturated fat intake demonstrated a significant intervention effect, with a highly significant time × group interaction (<0 0.001) due to a significant increase in saturated fat intake in the control group and a significant decrease in the intervention group (−9.24, *p* ≤0.001).

### 3.4. Knowledge, Attitudes, and Practices (KAP) Outcomes

Paired-samples *t*-tests, a type of inferential statistic used to determine if there is a significant difference between the means of two groups, were used to compare within-group differences. Repeated measures ANCOVA, a statistical technique used to compare the means of more than two groups that are related, was used to determine differences between groups over time, as shown in Table 4. In the control group, the results of the paired *t*-test showed no statistically significant differences in relation to the three measured outcomes. However, there was a slight increase in knowledge score between baseline (mean = 51.93, SD = 11.10) and post-intervention (mean = 53.68, SD = 14.00), *t* (94) = −1.30, *p*-value = 0.198, with a small effect size (Cohen’s d = −0.13). Similarly, no significant changes were observed in attitude or practice scores, *t* (94) = −1.78, *p*-value = 0.079, *d* = −0.18; and *t* (94) = −0.99, *p*-value = 0.708, *d* = −0.10, respectively. On the other hand, there was statistically significant improvement in knowledge score among students in the intervention group (from 44.77 ± 16.25 to 67.08 ± 18.44), *t* (120) = −13.03, *p*-value ≤ 0.001, with a large effect size (Cohen’s *d* = −1.18). However, there were no statistically significant changes in attitude and practice scores *t* (120) = −0.47, *p*-value = 0.643, *d* = −0.04; *t* (120) = 0.38, *p*-value = 0.324, *d* = +0.03, respectively. In order to compare the change in mean over time between the two groups (intervention and control groups), a repeated measures ANCOVA was carried out on each of the outcome variables. For knowledge, a significant main effect of time was observed, F(1, 214) = 111.998, *p*-value ≤ 0.001, along with a significant time × group interaction, F(1, 214) = 81.723, *p*-value ≤ 0.001. This interaction indicates that knowledge improvements were significantly greater in the intervention group (mean change = 22.31) compared to the control group (mean change = 0.198). For attitude, the main effect of time was not significant, F(1, 214) = 1.80, *p*-value = 0.182, and the time × group interaction was also non-significant, F(1, 214) = 0.39, *p*-value = 0.535. Both groups exhibited minor changes that were not statistically meaningful. Similarly, for practice, no significant time effect was found, F(1, 214) = 0.20, *p*-value = 0.656, and the interaction between time and group was non-significant, F(1, 214) = 0.93, *p*-value = 0.336.

## 4. Discussion

The current study evaluated the effectiveness of a five-month school-based vegetable gardening and nutritional education intervention on various parameters, including body composition, dietary intake, and KAP, for 4th–6th-grade students. The findings reveal a complex pattern of intervention effects that both support and challenge the existing literature on previous school gardening interventions [40]. To the best of our knowledge, this is the first comprehensive evaluation of the school gardening and nutrition education program initiative conducted in the Middle East. This intervention achieved improvements in children’s health outcomes, providing evidence for the effectiveness of implementing such interventions locally and in the region. Obesity affects all age groups, including children, and genders. It is influenced by dietary intake, physical activity, metabolism, genetics, and socioeconomic factors [65]. These results of our study are of importance given the rising prevalence of childhood obesity in Jordan and the urgent need for evidence-based interventions that can be incorporated into educational systems. The multifactorial nature of childhood obesity, which involves dietary intake, physical activity, metabolism, genetics, and socioeconomic factors, necessitates comprehensive intervention approaches that address multiple determinants simultaneously [65]. Our school-based gardening intervention specifically targeted dietary behaviors and nutrition knowledge as key modifiable factors in this complex obesity prevention framework.

### 4.1. Body Composition and Anthropometric Outcomes

Remarkable and clinically significant improvements in body composition parameters were achieved in response to the intervention. The intervention group demonstrated substantial enhancements in BMI (with a significant reduction of −1.57 kg/m^2^), BMI z-score (−0.37), and weight (−1.88 kg), while the control group showed minor increases in these measures (Table 2). These findings are notable, as previous meta-analyses and reviews have shown mixed results on the effectiveness of school gardening on health and no significant effect on BMI was reported, indicating that this intervention was well-tailored for our study group [43,66]. The baseline imbalances in gender distribution and height between groups, although statistically significant, were appropriately controlled for in the ANCOVA analyses. The significant time × group interaction effects observed across the anthropometric measures suggest that this intervention was effective in promoting healthy weight management. Our results align with recent evidence from a research group that demonstrated school-based gardening interventions can improve metabolic parameters in children, including reductions in LDL cholesterol [26]. These results regarding anthropometric measurements suggest using similar models in other schools is a promising strategy to prevent childhood obesity. The active involvement of children in food production, which were at least similar to the findings of other studies on school gardening interventions, also encouraged familiarity with vegetables, a lower degree of food neophobia, and self-efficacy in healthier food choices [1,6,59,67]. The school garden was also a learning ecosystem, strengthening nutrition, biology, and environmental responsibility cross-disciplinary knowledge.

### 4.2. Dietary Intake and Nutritional Outcomes

The intervention successfully changed children’s dietary patterns, achieving significant improvements in overall diet quality, demonstrating the impact of the intervention on nutritional health. The intervention group showed significant reductions in total energy intake (−145.35 kcal), compared to increases in the control group, suggesting improved dietary quality. This finding is of importance for addressing childhood obesity in Jordan, where excessive caloric intake has become a growing concern [53].

The intervention led to a significant improvement in dietary fiber intake (+2.36 g/day) and a substantial reduction in the consumption of saturated fat (−9.24 g/day) in the intervention group. These improvements represent clinically significant changes that align with previous systematic review evidence showing that school-garden-based programs can positively impact children’s dietary fiber intake and overall nutritional quality [40]. The reduction in saturated fat is important for the promotion of cardiovascular health among Jordanian children and could have long-term implications for reducing chronic disease risk in the Jordanian population. Furthermore, this intervention achieved significant improvements in potassium intake and reduced saturated fat consumption, meaning the intervention influenced overall diet quality. These nutritional improvements provide evidence that culturally adapted gardening interventions may promote healthier eating patterns among Jordanian children. However, while not reaching statistical significance, the intervention group demonstrated an improved trend in several essential nutrients compared to baseline. The changes were noted in vitamin C intake (83.55 to 98.67 mg/day, +15.12 mg) and vitamin D consumption (8.30 to 14.46 mcg/day, +6.16 mcg). However, several nutrients showed no significant improvements, including iron intake (intervention: −6.13 mg/day vs. control: −19.52 mg/day, *p*-value = 0.402) and calcium intake (intervention: +15.4 mg/day vs. control: −34.75 mg/day, *p*-value = 0.775). The lack of significant improvements in these micronutrients may reflect the intervention’s primary focus on vegetable consumption rather than comprehensive dietary modification, suggesting that future programs should consider broader nutritional targets to achieve more comprehensive micronutrient improvements.

In this study, vegetable intake frequency within groups demonstrated a significant change in time × group interaction. The intervention group demonstrated a modest increase from 2.7 to 2.9 per day pre- vs. post-intervention. When compared to the control group, vegetable intake had slightly decreased by the conclusion of the study. The existing literature suggests that some school-garden-based programs improve the consumption of fruits and vegetables in interventions that lasted 12–28 weeks [7,40]. This could be explained by the use of frequency measures rather than portion sizes, which may have limited our ability to detect significant changes in intake. Previous studies showed the importance of robust dietary assessment methods, with some research showing that positive results may be attributable to measurement limitations rather than the true effect of interventions [7]. Furthermore, evidence indicates that younger children at preschool and primary school age are more responsive to similar education programs and interventions, as they are more adaptable to changes.

### 4.3. Knowledge, Attitudes, and Practices (KAP)

The intervention was successful in improving children’s nutritional knowledge, demonstrating the educational potential of hands-on gardening interventions in Jordanian schools. Nutritional knowledge demonstrated the strongest improvement, with the intervention group achieving a substantial increase of +22.31 points, compared to +1.75 points in the control group. Notably, the intervention group had lower baseline knowledge scores (44.77 ± 16.25) compared to the control group (51.93 ± 11.10), yet achieved significantly greater improvements. This pattern suggests that children with limited prior nutritional knowledge may be more receptive to new information and behavioral change strategies, supporting the effectiveness of early educational interventions in populations with knowledge gaps [31]. Our findings support the educational component of this study and align with a previous systematic review showing that school-garden-based programs are effective in improving nutrition, gardening, and science education for children [68]. The hands-on nature of gardening activities combined with nutrition education has been shown to strengthen knowledge regarding healthy diets [69]. Active participation in school gardening activities, along with nutritional education in the school curriculum, strengthened children’s knowledge regarding horticulture and healthy eating habits [40]. Despite the significant gains in knowledge, the intervention did not significantly improve attitudes or practices related to vegetable consumption. This could be explained by the effect of the home environment, as this intervention does not address determinants such as the availability of food at home and family feeding practices. Previous research has shown that home fruit and vegetable availability is an important factor that affects a child’s food choice; if the food is not readily available at home, children cannot transfer their knowledge into practice [40]. Several factors may explain the limited improvements in attitudes and practices despite significant knowledge gains. The Theory of Planned Behavior suggests that knowledge alone is insufficient to modify behavior without addressing social norms and perceived control [70]. The five-month intervention duration may have been adequate for knowledge acquisition but insufficient for attitude crystallization and habit formation, which typically require 6–12 months [71]. The absence of family engagement represents a critical limitation, as parents serve as primary food gatekeepers and role models [72]. Additionally, home food environments and socioeconomic factors may create barriers to translating knowledge into practice [73]. Future interventions should incorporate multi-level approaches including parent education components and community partnerships to bridge the knowledge–practice gap [74]. The observed pattern of greater knowledge gains in students with a lower baseline knowledge suggests that targeting populations with limited prior nutrition education may yield more pronounced educational outcomes, though this requires confirmation through larger randomized trials.

### 4.4. Leading Study Establishing Foundation for Regional Implementation

As the first comprehensive school gardening interventional study conducted in Jordan, our research establishes baseline evidence for the effectiveness of similar programs in regional and Middle Eastern cultural and educational contexts. The positive outcomes of this study show that this program can be implemented and adapted within the Jordanian educational system in other schools and regions. The multiple significant improvements achieved across anthropometric measurements, dietary quality, and knowledge position this study as a regional achievement in promoting childhood health. The results support the effectiveness of multi-component school garden programs; such programs should be compared with other interventions. Evidence suggests that integrated school-based gardening programs that combine gardening with classroom lessons, nutrition education, and hands-on experience is more promising than single-component interventions [40,75]. The school garden was also a learning ecosystem, strengthening nutrition, biology, and environmental responsibility cross-disciplinary knowledge.

### 4.5. Strength and Limitations

This pre–post design is a controlled quasi-experimental design with a demographically matched control school, a prior power calculation, and field standard procedures, which improve internal validity. Trained personnel were used in collecting objective anthropometric data, which was then analyzed using the WHO 2025 [60] growth reference (Anthroplus), thus making it possible to provide age- and sex-standardized results. The intervention was a multi-component one (including weekly gardening, guided tasting, and classroom-based nutrition education) taking place over five months and was culturally adapted. Systematic reviews and empirical trials support this approach, showing that school garden programs have the potential to improve diet quality and the intake of vegetables, although effects on BMI are not always apparent. Dietary intake was assessed using two unannounced, non-consecutive 24 h dietary recalls at each time point using the USDA Automated Multiple-Pass Method—a validated protocol that minimizes recall bias and is consistent with doubly labeled water recalls. A linguistic translation and a thorough psychometric test were performed on the KAP instrument, including internal consistency, test–retest reliability, and principal component analysis using Kaiser–Meyer–Olkin (KMO) tests and Bartlett’s test of sphericity. The reporting followed the Transparent Reporting of Evaluations with Nonrandomized Designs (TREND) instructions, thus improving the level of transparency and reproducibility of the study. Finally, this research provides the first controlled empirical data originating in Jordan—an underrepresented area of school-garden research—therefore supporting the contextual relevance of school-based gardening in addition to nutritional education in Middle Eastern contexts. However, using frequency measures for fruit and vegetable intake may have limited our ability to detect meaningful changes in consumption. Previous studies noted that measurement methods significantly influence the ability to detect intervention effects, with some studies showing positive results due to limitations in measurement tools rather than true intervention effects [76]. The significant baseline imbalances in gender distribution (*p*-value ≤ 0.001) and height (*p*-value ≤ 0.001) between groups represent a limitation of the quasi-experimental design. However, these differences were appropriately controlled through covariate adjustments in all analyses, and the consistent significant time × group interactions across multiple outcomes support the robustness of our intervention effects.

Additionally, the lack of parental involvement to reinforce and support the efforts made in school may have been a limitation of the approach. Parents are known to play a fundamental role in the development and achievement of children and, according to social learning theory, children learn from and mimic the behavior of others through observation. Since children spend a good amount of time with their family, their food choices, eating behaviors, and eating attitudes are hugely influenced by their parents [9]. Finally, the reliance on self-reported dietary intake through 24 h recalls, despite using the validated USDA Automated Multiple-Pass Method, may introduce recall bias and social desirability effects that could influence dietary reporting accuracy. The absence of family engagement represents a critical limitation, as home food environments and parental practices are the primary determinants of children’s dietary behaviors [9]. This may explain why knowledge improvements did not translate into significant attitude and practice changes, as children cannot implement learned behaviors without supportive home environments and food availability [9].

### 4.6. Future Directions and Recommendations

#### 4.6.1. Program Sustainability and Scalability

The sustainability of school gardening programs depends on institutional integration and resource allocation. Evidence from similar interventions suggests that programs require ongoing administrative support, teacher training, and infrastructure maintenance to achieve a lasting impact [1,7]. Our intervention model demonstrates adaptability to Middle Eastern contexts through culturally appropriate crop selection and Arabic educational materials, supporting its potential scalability across diverse socioeconomic settings within the region. However, successful scaling requires addressing resource disparities, as schools in lower-income areas may need additional funding support and community partnerships. The significant improvements in body composition and knowledge observed in this study provide evidence for policy-makers to consider integrating school gardening programs into national childhood obesity prevention strategies, particularly given Jordan’s 28% childhood overweight/obesity prevalence [7].

#### 4.6.2. Future Implementation Strategies

To ensure the success of a similar study, it is recommended to involve parents by offering them practical advice on fostering children’s preferences toward healthier foods and educating them about the negative impact of forced feeding practice; this will help to establish positive parental role models [40]. Another recommended strategy is to include practical advice on increasing the children’s willingness to consume unfamiliar foods, mainly fruits and vegetables. Future school-garden-based programs should adopt a multi-level approach involving school, home, and community environments to maximize intervention scope and impact [77]. This may include incorporating food service into the intervention, such as setting up school salad bars using garden-grown crops and adding fruits as snacks in school cafeterias. Future programs are recommended to consider establishing relationships with local farmers and community gardens and to promote locally grown produce to students and their families to maximize exposure to fruits and vegetables while helping the community. The significant improvements in body composition and knowledge suggest the potential for a lasting impact. However, longitudinal studies are needed to assess the sustainability of these effects and determine optimal intervention characteristics for long-term behavioral changes.

## 5. Conclusions

This study demonstrates that well-designed school-based gardening interventions can achieve significant improvements in body composition and nutritional knowledge in children. While challenges remain in translating knowledge gains into sustained dietary behavior changes, this intervention could successfully address the childhood obesity markers that represent an important contribution to school-based health promotion efforts [1]. Our findings support the continued development of multi-component school gardening programs, with emphasis to incorporating family engagement and extending intervention effects beyond the school environment. The significant improvement in BMI and weight status, along with nutritional knowledge, provides evidence for the potential of school gardening interventions to address childhood obesity while building lifelong healthy eating behaviors. These results suggest continued investment in similar programs with attention to implementation and methodological lessons learned from this trial.

## Figures and Tables

**Table 1:** Baseline differences in demographic and clinical characteristics between intervention and control groups.

	All Participants	Control (*n* = 95)	Intervention (*n* = 121)	*p*-Value
Age	10.18 ± 1.22	10.35 ± 1.17	10.05 ± 1.24	0.073
Gender *				
Male	88 (100.0%)	22 (25.0%)	66 (75.0%)	≤0.001
Female	128 (100%)	73 (57.0%)	55 (43.0%)	
Weight	42.17 ± 2.62	41.80 ± 3.12	42.45 ± 2.11	0.072
Height	131.78 ± 4.33	133.64 ± 5.07	130.32 ± 2.94	≤0.001

Continuous variables were compared using independent-samples *t*-tests, while categorical variables were compared using chi-square (χ^2^) tests. All tests were two-tailed with a significance level of *p*-value ≤ 0.05. Data are presented as mean ± standard deviation (SD) and * frequencies (%).

**Table 2:** Changes in anthropometric and dietary outcomes among primary school children before and after the gardening intervention.

Anthropometric Variables	Control (*n* = 95)				Intervention (*n* = 121)					
	Baseline (Mean ± SD)	Post-Intervention (Mean ± SD)	Absolute Change	Within Group	Baseline (Mean ± SD)	Post-Intervention (Mean ± SD)	Absolute Change	Within Group	Time	Time × Group Interaction *p*-Value
				*p*-Value				*p*-Value	*p*-Value	*p*-Value
Height (cm)	133.64 ± 5.07	134.92 ± 4.93	1.28	<0.001	130.32 ± 2.94	131.61 ± 3.16	1.29	<0.001	0.941	<0.001
Weight (kg)	41.81 ± 3.12	42.67 ± 3.06	0.86	<0.001	42.45 ± 2.11	40.57 ± 2.14	1.88	<0.001	0.040	<0.001
BMI (kg/m^2^)	23.47 ± 2.07	23.50 ± 1.99	0.03	<0.001	25.02 ± 1.46	23.45 ± 1.45	1.57	<0.001	0.001	<0.001
BMI z-score	−0.45 ± 1.08	0.02 ± 1.17	0.47	0.050	0.36 ± 0.76	−0.01 ± 0.85	0.37	<0.001	0.003	<0.001
BMI Percentile	96.29 ± 6.16	96.51 ± 4.71	0.22	0.072	97.89 ± 1.16	97.10 ± 2.16	0.79	0.002	0.031	0.002
Vegetable intake (freq/d)	2.5 ± 1.4	2.4 ± 1.5	0.1	0.886	2.7 ± 1.6	2.9 ± 1.5	0.2	0.49	0.45	0.003
Fruit intake (freq/d)	2.4 ± 1.7	2.6 ± 1.8	0.2	0.93	2.7 ± 1.9	2.8 ± 1.9	0.1	0.49	0.50	0.184

Values are presented as mean ± standard deviation (SD). Within-group comparisons were conducted using paired-sample *t*-tests. Between-group differences over time were assessed using repeated measures ANCOVA. Vegetable and fruit intake are reported as frequency per day (times/day). All tests were two-tailed with a significance level of *p*-value ≤ 0.05. Absolute change values are reported as |post–baseline| without minus signs; decreases are interpreted accordingly.

**Table 3:** Changes in energy, macronutrient, and micronutrient intake among primary school children before and after the gardening intervention.

	Control (*n* = 95)				Intervention (*n* = 121)					
Dietary Components	Baseline (Mean ± SD)	Post-Intervention Mean ± SD	Absolute Change Mean	Within Group	Baseline (Mean ± SD)	Post-Intervention Mean ± SD	Absolute Change Mean	Within Group	Time	Time × Group Interaction *p*-Value
				*p*-Value				*p*-Value	*p*-Value	*p*-Value
Macronutrients										
Total Energy, kcal	1519.72 ± 398.35	1571.97 ± 488.64	52.25	0.086	1622.34 ± 450.71	1476.99 ± 421.27	145.35	≤0.001	0.066	≤0.001
Protein, g/day	56.99 ± 19.58	52.31 ± 25.55	4.68	0.085	60.12 ± 26.66	52.91 ± 26.42	7.21	0.023	0.006	0.553
Carbohydrate, g/day	205.26 ± 60.83	202.26 ± 65.09	3.00	0.642	210.04 ± 68.72	195.15 ± 77.65	14.89	0.076	0.105	0.280
Fat, g/day	52.49 ± 21.94	58.73 ± 33.49	6.24	0.034	60.85 ± 29.34	56.60 ± 32.62	4.25	0.216	0.668	0.025
Shortfall Micronutrients										
Vitamin A, mcg/day	308.68 ± 725.02	433.62 ± 826.46	124.94	0.665	293.85 ± 430.06	435.19 ± 953.69	141.34	0.352	0.077	0.900
Vitamin E, mg/day	7.01 ± 22.02	6.03 ± 8.65	0.98	0.931	5.03 ± 7.87	6.74 ± 20.49	1.71	0.000	0.789	0.324
Vitamin C, mg/day	100.34 ± 139.62	84.19 ± 93.80	16.15	0.605	83.55 ± 75.35	98.67 ± 111.36	15.12	0.836	0.960	0.134
Magnesium, mg/day	11.21 ± 5.45	10.20 ± 6.30	1.01	0.146	10.14 ± 5.16	10.56 ± 9.09	0.42	0.000	0.189	0.490
Iron, mg/day	136.38 ± 114.60	116.86 ± 89.29	19.52	0.493	123.50 ± 121.25	117.37 ± 130.81	6.13	0.402	0.616	0.226
Micronutrients of Public Health Concern										
Dietary Fiber, g/day	14.69 ± 7.47	13.45 ± 8.79	1.24	0.228	13.26 ± 5.47	15.62 ± 9.75	2.36	0.030	0.453	0.017
Calcium, mg/day	640.44 ± 340.05	605.69 ± 468.80	4.75	0.528	593.80 ± 321.71	609.20 ± 438.85	15.4	0.775	0.794	0.498
Vitamin D (calciferol), mcg/day	7.21 ± 61.86	10.42 ± 45.91	3.21	0.691	8.30 ± 57.54	14.46 ± 73.31	6.16	0.317	0.352	0.766
Potassium, mg/day	1364.83 ± 625.04	1036.73 ± 682.89	328.1	≤0.001	1223.66 ± 850.44	1243.53 ± 699.00	19.87	0.840	0.011	0.023
Nutrients to Reduce or Limit										
Sugar, g/day	64.02 ± 34.25	63.08 ± 41.85	0.94	0.86	71.27 ± 44.05	61.54 ± 45.88	9.73	0.08	0.17	0.25
Saturated Fat g/day	15.69 ± 9.74	20.41 ± 19.26	4.70	0.025	25.25 ± 21.76	16.01 ± 13.39	9.24	≤0.001	0.158	≤0.001
Sodium, mg/day	1409.48 ± 827.95	1871.41 ± 1406.31	461.93	0.007	2022.38 ± 1250.63	2306.42 ± 1959.64	284.04	0.192	0.010	0.534

Values are presented as mean ± standard deviation (SD). Within-group comparisons were conducted using paired-sample *t*-tests. Between-group differences over time were assessed using repeated measures ANCOVA. All tests were two-tailed with a significance level of *p*-value ≤ 0.05. Absolute change values are reported as |post–baseline| without minus signs; decreases are interpreted accordingly.

**Table 4:** Changes in knowledge, attitude, and practice (KAP) scores related to vegetable consumption among primary school children before and after the gardening intervention.

KAP Variables	Control (*n* = 95)				Intervention (*n* = 121)				Time	Time × Group Interaction *p*-Value
	Baseline (Mean ± SD)	Post-Intervention (Mean ± SD)	Absolute Change	Within Group	Baseline (Mean ± SD)	Post-Intervention (Mean ± SD)	Absolute Change Mean	Within Group		
				*p*-Value				*p*-Value	*p*-Value	*p*-Value
Knowledge score	51.93 ± 11.10	53.68 ± 14.00	1.75	0.198	44.77 ± 16.25	67.08 ± 18.44	22.31	<0.001	<0.001	<0.001
Attitude score	91.93 ± 13.29	94.56 ± 10.15	2.63	0.079	84.44 ± 21.49	85.40 ± 22.42	0.96	0.643	0.182	0.535
Practice score	74.24 ± 25.18	73.28 ± 24.20	−0.96	0.708	81.93 ± 19.85	84.56 ± 18.71	2.63	0.324	0.656	0.336

Values are presented as mean ± standard deviation (SD). Within-group comparisons were conducted using paired-sample *t*-tests. Between-group differences over time were assessed using repeated measures ANCOVA. All tests were two-tailed with a significance level of *p*-value ≤ 0.05. Absolute change values are reported as |post–baseline| without minus signs; decreases are interpreted accordingly.

## Data Availability

The information that supports the findings of the research can be received from the author upon reasonable request due to the privacy of the participants.

## Supplementary Materials

The following supporting information can be downloaded at: https://www.mdpi.com/article/10.3390/nu17193133/s1.
