# Supplementary material for: A School-Based Five-Month Gardening Intervention Improves Vegetable Intake, BMI, and Nutrition Knowledge in Primary School Children: A Controlled Quasi-Experimental Trial
Source: Nutrients. 2025 Sep 30;17(19):3133. doi: 10.3390/nu17193133 (PMC12526235; doi:10.3390/nu17193133)
Supplement: Supplementary file 1 [file nutrients-17-03133-s001.zip › nutrients-3879108-supplementary.pdf]

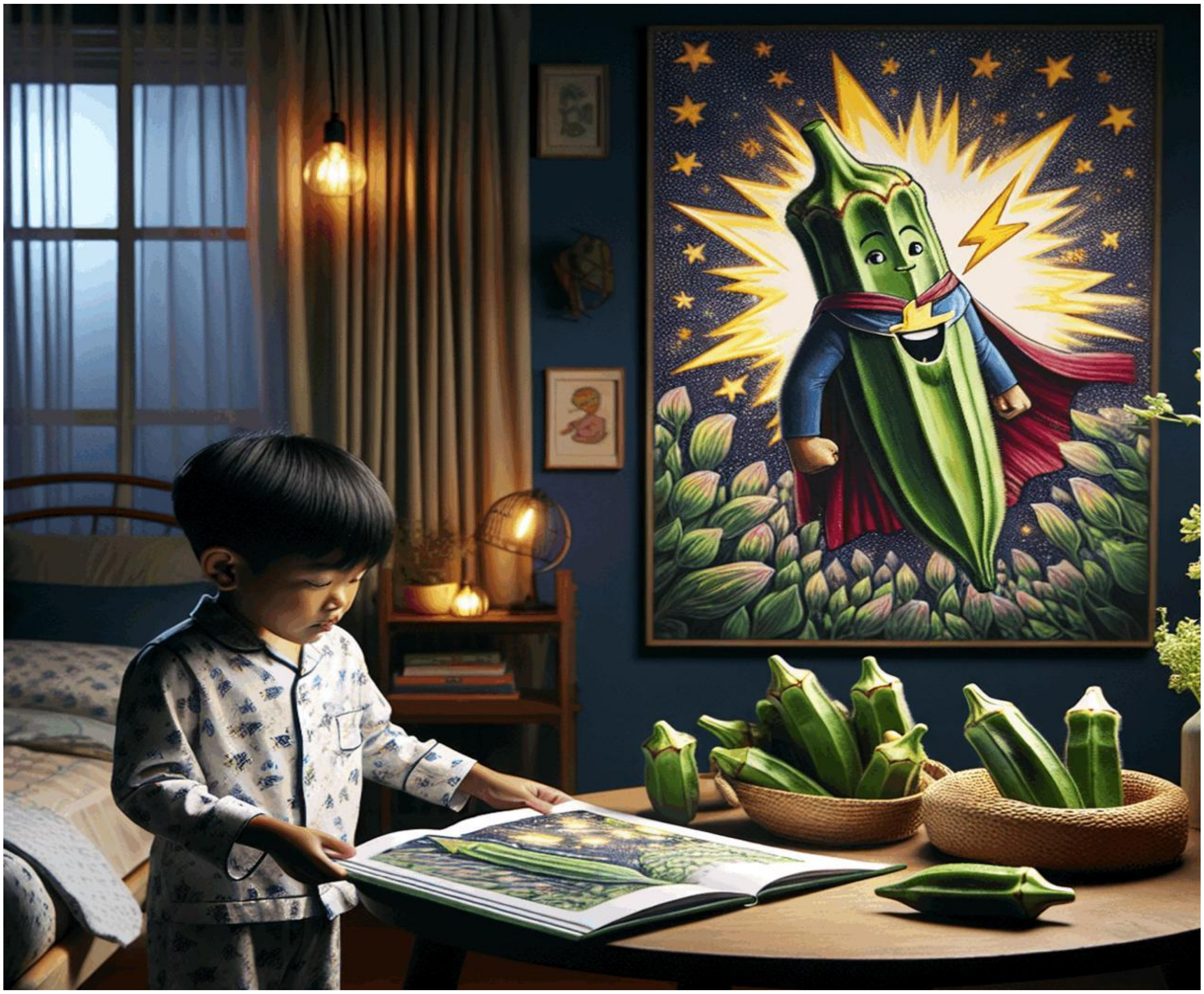

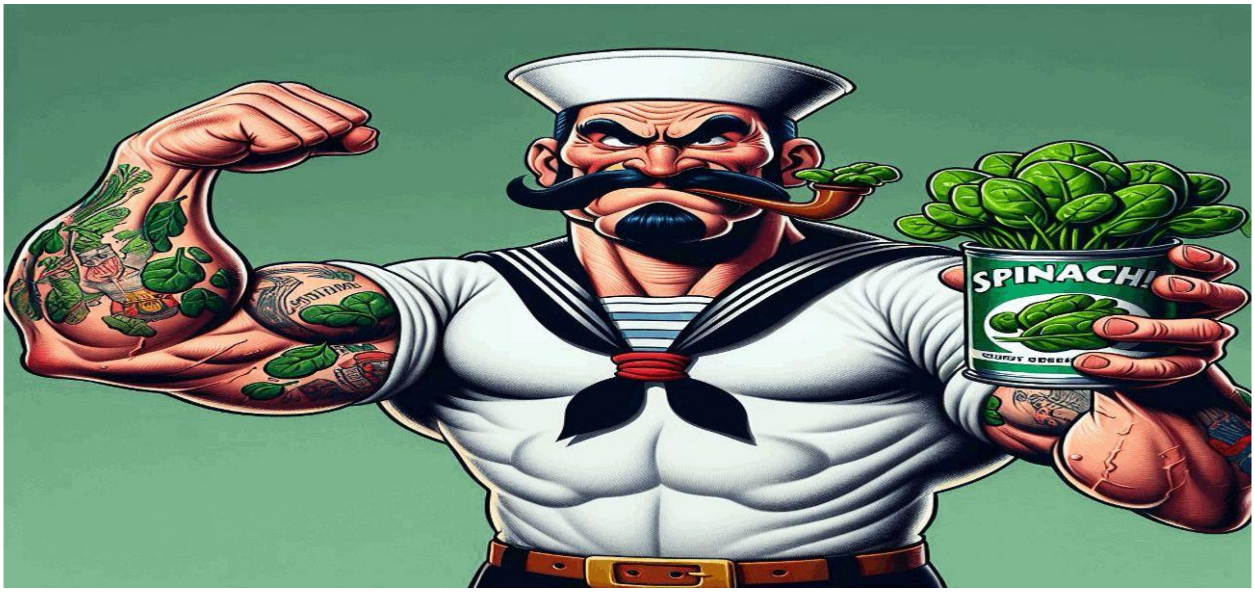
SPINACH!

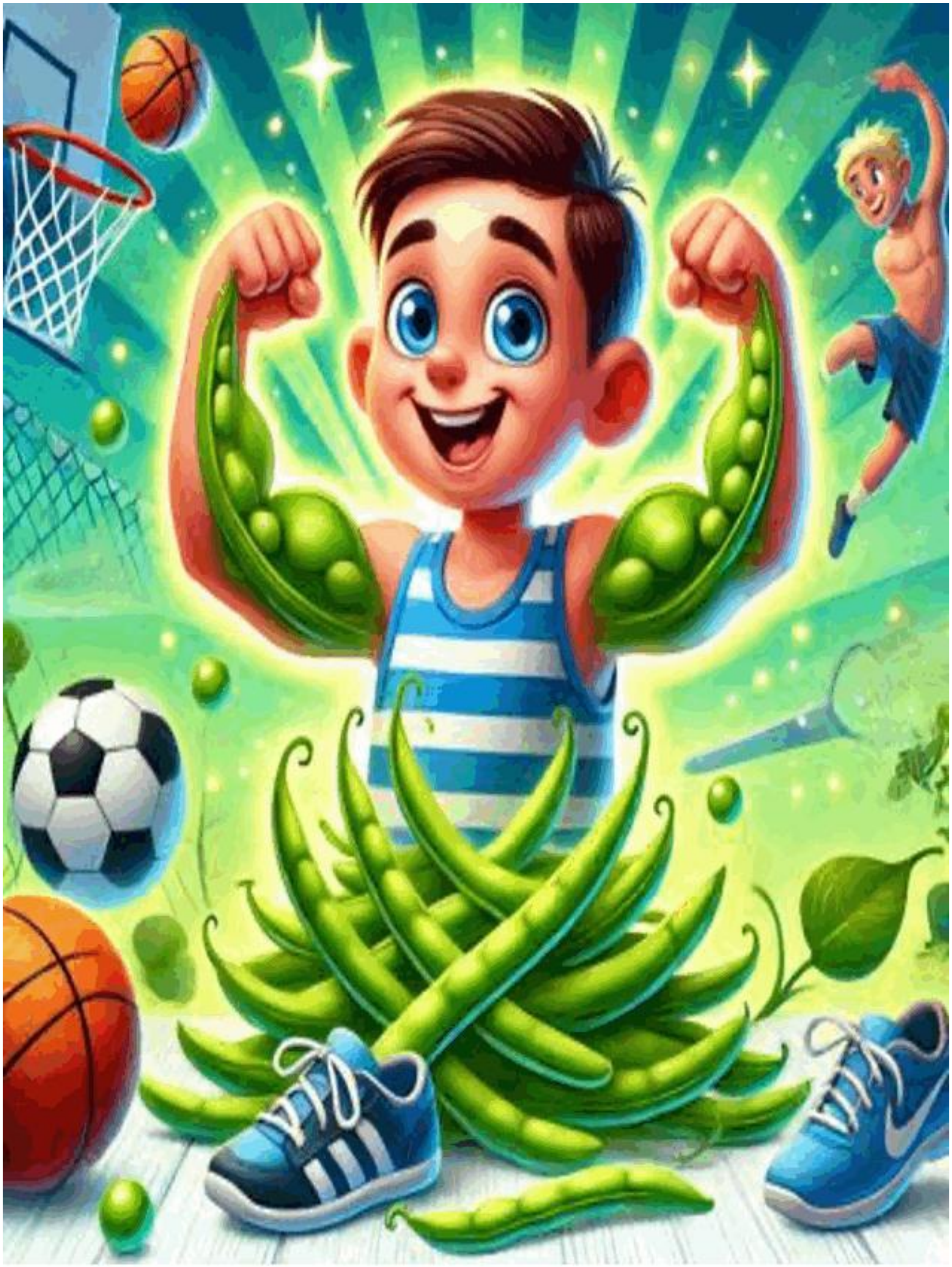

	Fun Vegetable Potential Benefits for Children (Massages)
1.	Carrots make your eyes strong like Superman.
2.	Spinach gives you strength like brave Popeye the sailor.
3.	Did you know that broccoli looks like little trees? When you eat it, it provides you with stronger bones and muscles.
4.	Red Tomatoes make your heart happy and healthy.
5.	Sweet Potatoes help your body fight disease like a brave soldier.
6.	Cucumbers contain water that makes your skin fresh and beautiful.
7.	Green lettuce helps protect your teeth and makes them strong.
8.	Sweet peppers are colorful and beautiful, providing you with important vitamins.
9.	White cauliflower helps your bones grow healthily.
10.	Green peas are like little balls full of energy.
11.	When you eat vegetables, you become smarter at school.
12.	Zucchini helps you grow and provides strength.
13.	Eating vegetables makes you run faster on the playground.
14.	Purple eggplant protects your body from diseases.
15.	Green beans provide you with energy to play all day long.
16.	Cabbage makes your hair shiny and healthy.
17.	Red beets help strengthen your blood and increase your energy.
18.	Yellow corn strengthen eye and protect your body from diseases.
19.	Leafy vegetables make your brain work faster.
20.	Chard helps your muscles grow strong.
21.	Radishes help strengthen your immune system to fight germs.
22.	Eating Green onions maintains the health of your intestines.
23.	Beetroot makes your heart beat strongly like a hero.
24.	Okra helps your eyes see well.
25.	Turnips play a beneficial role in mitigating hepatic injury caused by diabetes.
26.	Artichokes help support your liver in cleaning your body.
27.	Asparagus offer protective benefits for the stomach and support immune system.
28.	Celery helps you breathe better.
29.	Green fava beans built strong muscles.
30.	Green basil makes your food delicious and beneficial for your health.
